# Simultaneous laparoscopic adrenal and gallbladder surgery: a case of combined minimally invasive success

**DOI:** 10.1093/jscr/rjag184

**Published:** 2026-03-22

**Authors:** Selçuk Mercan, Osman Anıl Savaş, Amir Mahdi Akbari, Mehrdad Sheikhvatan

**Affiliations:** Faculty of Medicine, Department of General Surgery, İstanbul Okan University, İstanbul, Turkey; Faculty of Medicine, Department of General Surgery, İstanbul Okan University, İstanbul, Turkey; Faculty of Medicine, Department of General Surgery, İstanbul Okan University, İstanbul, Turkey; Faculty of Medicine, Department of General Surgery, İstanbul Okan University, İstanbul, Turkey

**Keywords:** adrenalectomy, cholecystectomy, cholecystitis, cholelithiasis, laparoscopic surgery

## Abstract

Concomitant indications for adrenalectomy and cholecystectomy are rare, and simultaneous laparoscopic performance of both procedures is infrequently reported. Combined surgery may reduce anesthetic exposure, hospital stay, and recovery time. A 57-year-old male was admitted with intermittent pain in the right upper quadrant and abdominal distension. Imaging confirmed cholelithiasis with chronic cholecystitis, along with an incidental, nonfunctional 8-cm right adrenal mass. The patient underwent an uneventful simultaneous laparoscopic right adrenalectomy and cholecystectomy. Histopathology confirmed chronic cholecystitis with cholelithiasis and a benign adrenal cavernous hemangioma. The postoperative course was uneventful. This case demonstrates that laparoscopic adrenalectomy combined with simultaneous cholecystectomy can be safely performed in selected patients, this approach enables efficient management of two intra-abdominal pathologies while minimizing surgical morbidity.

## Introduction

The simultaneous presence of adrenal masses and biliary pathology poses a particular challenge in diagnosis and treatment, especially when both conditions require surgical intervention. Gallstone disease is one of the most common causes of abdominal symptoms worldwide and a frequent indication for laparoscopic cholecystectomy [1]. Conversely, adrenal masses are often discovered incidentally during imaging performed for unrelated reasons [2]. Although most adrenal incidentalomas are non-functioning larger lesions typically necessitate surgical removal due to the risk of malignancy and other serious complications [3].

Combining laparoscopic cholecystectomy and laparoscopic adrenalectomy is uncommon and requires meticulous planning and highly specialized advanced laparoscopic techniques and the appropriate patient [4]. While the safety, feasibility and efficacy of concomitant minimally invasive procedures have been demonstrated in selected cases, reports indicate this approach remains underdeveloped. The decision to perform concomitant procedures should weigh the benefits of reduced anesthesia exposure against the drawbacks of longer operating times and varying recovery periods [5].

A 57-year-old male presented with symptomatic cholelithiasis and was incidentally found to have a large right adrenal mass. He subsequently underwent a successful simultaneous laparoscopic right adrenalectomy and laparoscopic cholecystectomy. This case offers valuable insights into both sound clinical reasoning and the technical advantages of combining these procedures into a single operative session.

## Case report

A 57-year-old male with no significant medical or family history, presented with intermittent abdominal pain and distention. The pain was dull, typically occurred after meals. It was not associated with nausea, vomiting, jaundice, fever, palpitations, headaches, or sweating. Physical examination revealed a soft abdomen with localized tenderness in the right upper quadrant. There were quadrant, but no palpable masses. Laboratory tests unremarkable. Imaging of the abdomen confirmed the presence of cholelithiasis and chronic cholecystitis. Additionally, an incidental finding revealed a large mass measuring 8 cm on the right adrenal gland. Because the mass on the adrenal gland and gallbladder were fairly large pathologically, surgical intervention was made.

**Figure 1:**
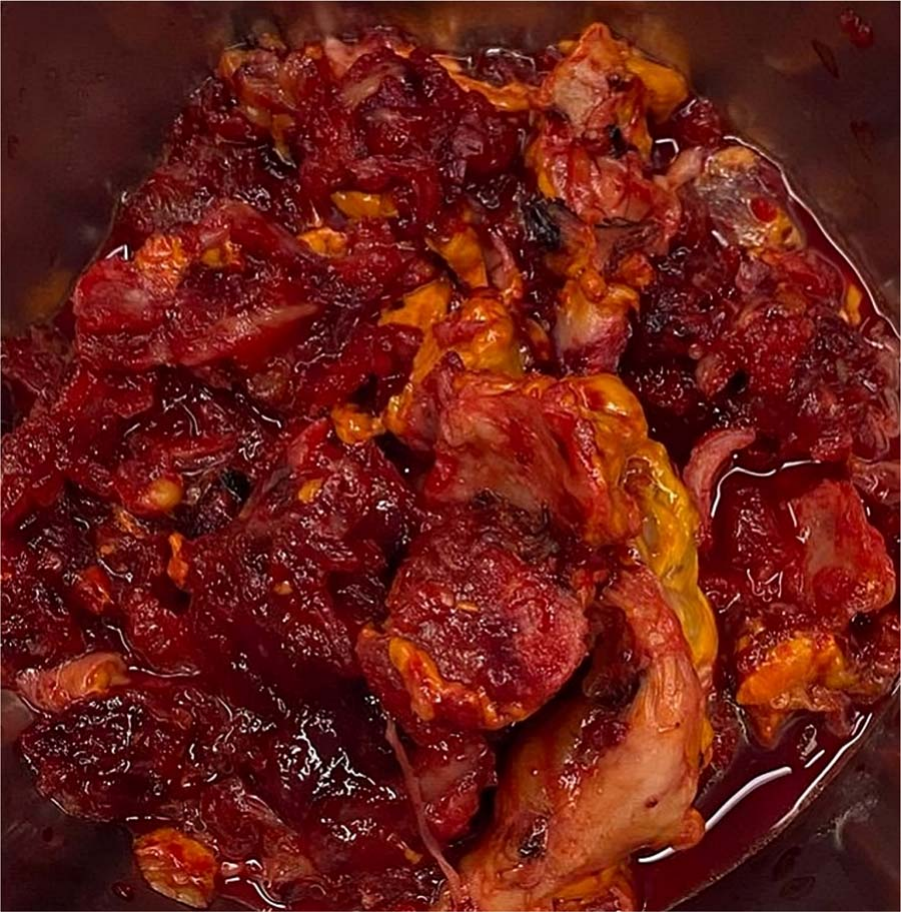
A specimen of adrenal gland extracted by laparoscopic adrenalectomy.

**Figure 2:**
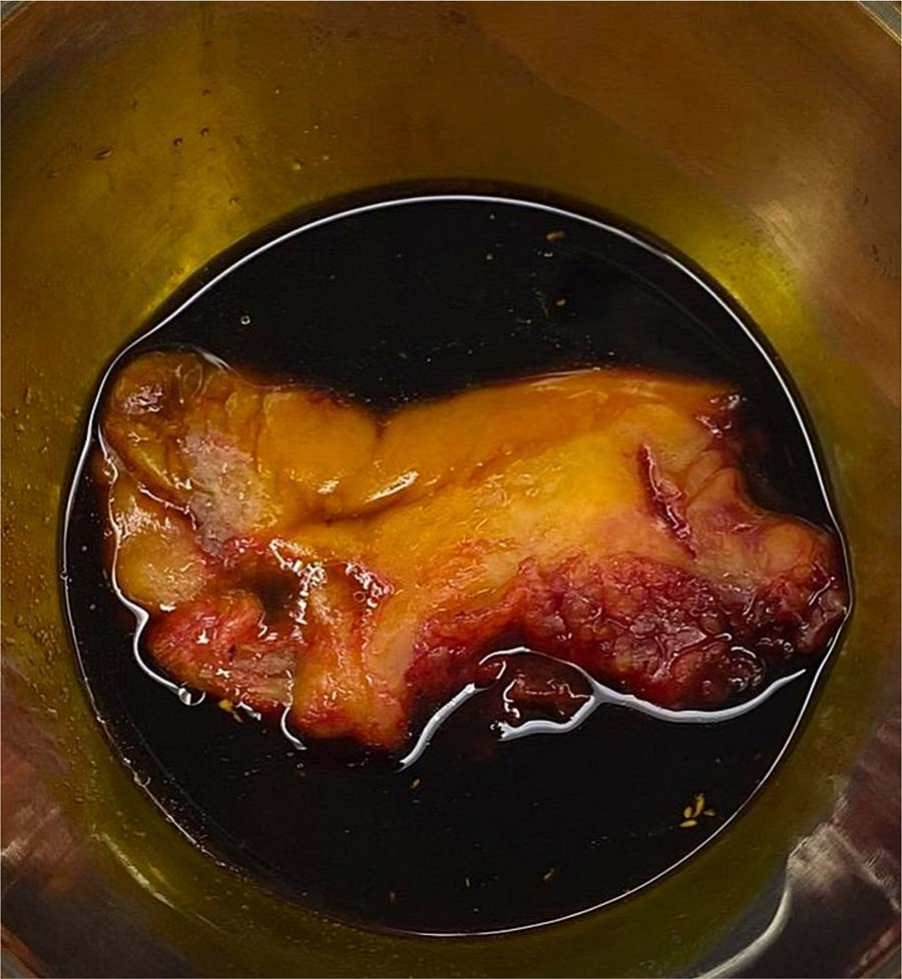
A specimen of gull bladder extracted by laparoscopic cholecystectomy.

The patient underwent a laparoscopic right adrenalectomy and laparoscopic cholecystectomy under general anesthesia. After establishing pneumoperitoneum, standard laparoscopic ports were inserted. The right adrenal gland was dissected using ligatures, and the adrenal vein was clipped and divided. The adrenal gland was then removed using an endobag (Fig. 1). The procedure continued with the laparoscopic cholecystectomy. During laparoscopic cholecystectomy, the cystic duct and cystic artery were identified, clipped and divided, followed by removal of gallbladder from the liver bed (Fig. 2). Hemostasis was confirmed no intraoperative complications occurred. Both procedures lasted ~115 min. Gross pathological examination revealed an 8.5 × 4 cm gallbladder with green mucosa and multiple gallstones, consistent with chronic cholecystitis. The adrenal specimen measured 11 × 9.25 × 2.5 cm and appeared as a gray-brown tissue fragment. Histologically, findings were consistent with chronic cholecystitis and cholesterolosis of the gallbladder, as well as a cavernous hemangioma with infarction, hyalinization, and dystrophic calcifications within the adrenal gland. Immunohistochemical staining was negative for PANCK and ACTH, showed weak and patchy staining for inhibin, and was positive for CD31 and CD34. These results suspected a diagnosis of adrenal hemangioma, excluding the previously considered diagnosis of malignant adrenal mass (C74.9). The patient’s postoperative condition remained stable, with a decrease in serohemorrhagic fluid. The surgical site was clean, and there were no issues with oral intake. The patient was discharged and scheduled for followed-up.

## Discussion

The simultaneous presence of symptomatic gallstone disease and an incidental adrenal mass represents a relatively uncommon clinical scenario that requires an integrated and personalized approach [1, 2]. Cholelithiasis frequently causes pain in the right upper quadrant; consequently laparoscopic cholecystectomy is widely regarded as the most effective treatment for symptomatic disease [6]. Conversely, adrenal masses are commonly discovered incidentally on imaging, usually performed for unrelated pathologies, and are often asymptomatic [7]. Although most adrenal lesions are small (4–6 mm) and non-functioning, current guidelines recommend surgical intervention for lesions larger than 4–6 cm due to an increased risk of malignancy and hemorrhage. In our case, the patient’s 8 cm right adrenal mass met the criteria for surgery [8].

The plan for concurrent laparoscopic right adrenalectomy and laparoscopic cholecystectomy represents an increasingly feasible and safe approach for combining these procedures. Although there has been a growing number of reports on simultaneous laparoscopic surgeries, including multiple procedures involving cholecystectomy, reports on combining adrenalectomy with cholecystectomy remain limited [9]. However, several benefits exist when completing these procedures in a single session. These include a shorter duration of exposure to general anesthesia, a reduced overall hospital stay, less cumulative postoperative pain, and a faster return to daily activities [10].

Careful planning is essential to undertake these two procedures. Laparoscopic adrenalectomy, especially on the right side, presents technical challenges due to its proximity to the inferior vena cava [11]. Performing both procedures simultaneously may increase the risk of complications because of the extended operative time [12]. Patient selection becomes an issue before undertaking these procedures. The patient should be stable, have a non-functioning adenoma or a well-optimized functioning adenoma, and have no contraindications to laparoscopic surgery [13].

The Justification for simultaneous surgery in this case included the patient’s excellent health status, the nonfunctional nature of the adrenal tumor, and the benefit of addressing two issues under a single general anesthetic. The surgery proceeded smoothly without complications. It appears that, with appropriate expertise, laparoscopic adrenalectomy combined with simultaneous cholecystectomy can be an equally successful option [14]. Similar documented cases in the medical literature support the feasibility of simultaneous procedures, particularly in high-volume laparoscopic centers [10].

This study contributes to the still limited but emerging literature by emphasizing the feasibility of simultaneous minimally invasive operations for multiple abdominal pathologies. It highlights the importance of personalized surgical planning to achieve optimal treatment outcomes for patients with multiple surgical conditions.

## Data Availability

All study data will be available to editorial board and readers upon request.
